# Tracheal Stenosis in Open Versus Percutaneous Tracheostomy

**DOI:** 10.7759/cureus.57075

**Published:** 2024-03-27

**Authors:** Darby L Keirns, Ajay K Rajan, Shirline H Wee, Isheeta S Govardhan, Dana N Eitan, Danielle B Dilsaver, Ian Ng, Marcus W Balters

**Affiliations:** 1 Department of Surgery, Creighton University School of Medicine, Phoenix, USA; 2 Department of Clinical Research and Public Health, Creighton University School of Medicine, Omaha, USA; 3 Department of Surgery, Creighton University School of Medicine, Omaha, USA

**Keywords:** tracheostomy complications, surgical tracheostomy, open tracheostomy, percutaneous tracheostomy, tracheal stenosis

## Abstract

Objective: This study aims to investigate if there is an increased risk of developing tracheal stenosis after tracheostomy with an open versus percutaneous tracheostomy.

Methods: The patient cohort included patients receiving open or percutaneous tracheostomies at Catholic Health Initiatives Midwest facilities from January 2017 to June 2023. The primary aim was to compare the differences in the risk of developing tracheal stenosis between open and percutaneous tracheostomy techniques. Between-technique differences in the risk of developing tracheal stenosis were assessed via a Cox proportional hazard model. To account for death precluding patients from developing tracheal stenosis, death was considered a competing risk.

Results: A total of 828 patients met inclusion criteria (61.7% open, 38.3% percutaneous); 2.5% (N = 21) developed tracheal stenosis. The median number of days to develop tracheal stenosis was 84 (interquartile range: 60 to 243, range: 6 to 739). Tracheal stenosis was more frequent in patients who received a percutaneous tracheostomy (percutaneous: 3.5% vs. open: 2.0%); however, the risk of developing tracheal stenosis was statistically similar between open and percutaneous techniques (HR: 2.05, 95% CI: 0.86-4.94, p = 0.108).

Conclusions: This study demonstrates no significant difference in the development of tracheal stenosis when performing an open versus a percutaneous tracheostomy. Tracheal stenosis is a long-term complication of tracheostomy and should not influence the decision about the surgical technique used.

## Introduction

A tracheostomy is a procedure performed to create an airway through the insertion of a tracheostomy tube into the neck [1]. It is estimated that over 100,000 tracheostomies are performed each year in the United States. The most common indications for tracheostomy include respiratory failure with prolonged mechanical ventilation, neurological insults, and airway obstruction [2]. The procedure has long been done as an open surgical procedure. However, in 1955, a new percutaneous technique was introduced, although it was not widely used until an additional dilatational technique was published in 1985 [3]. The standard open procedure is performed in the operating room and involves creating an incision along the neck to directly visualize and open the trachea for tube placement. The percutaneous procedure differs in that it is done at the bedside with a small neck incision and dilational placement of the tube via a modified Seldinger technique, sometimes with the guidance of a bronchoscope [1].

As the use of percutaneous tracheostomies has increased, studies have credited the percutaneous approach as a way to eliminate the use of an OR, decrease procedure time, reduce patient risk of transport, and therefore be more cost-effective [4,5]. Previous studies have compared the short-term complications between percutaneous and open tracheostomies, finding that the percutaneous technique may have a lower rate of complications such as perioperative and post-operative bleeding, post-operative infection, and the overall post-operative complication rate [6-7].

Long-term complications of a tracheostomy have also been described, such as tracheal stenosis. Tracheal stenosis develops in the setting of a tracheostomy due to irritation and trauma from the procedure and the presence of a tracheostomy tube, which leads to inflammation, abnormal wound healing, and the formation of granulation or scar tissue that can result in stenosis [8]. Tracheal stenosis has been reported to develop in anywhere from 0.6% to 22% of patients following tracheostomy and accounts for 28% of tracheostomy-related complications [9-10]. Patients may have symptoms such as trouble breathing, difficulty weaning from ventilation or decannulation, the need for additional procedures to address the stenosis, and an increased mortality risk [8,10-11]. Tracheal stenosis has been frequently found to be associated with prolonged mechanical ventilation [12-14]. Other reported factors associated with tracheal stenosis include increasing age, female sex, smoking, and comorbidities including diabetes, gastroesophageal reflux disease (GERD), chronic obstructive pulmonary disease (COPD), hypertension, and obesity [8,13,15-18].

Late complications of tracheostomy, such as tracheal stenosis, tend to be less understood than perioperative or early complications, as they are more challenging to study due to limitations in maintaining consistent follow-up [19]. When comparing tracheal stenosis seen in open versus percutaneous tracheostomies, the current literature presents inconsistent findings. Maccallum et al. [20] and Hill et al. [21] found a higher rate of tracheal stenosis in open procedures. Other studies, including Koitschev et al. [22] and Li et al. [13], found a higher rate of tracheal stenosis in percutaneous procedures. Further, Kettunen et al. [23] and de Kleijn et al. [24] demonstrated no significant difference between techniques. Many of these findings are limited by sample size, study timeframe, lack of follow-up, the subclinical nature of tracheal stenosis, and a small focus on tracheal stenosis amongst a wide range of complications.

Tracheal stenosis is a significant complication, and understanding the difference in outcomes and prevalence when comparing techniques is vital to making informed clinical decisions and sharing accurate risk factors with patients. Given the conflicting data in the literature, it is worthwhile to specifically investigate the long-term post-operative complications of tracheal stenosis in tracheostomy further and with a larger sample size. This study’s primary aim is to evaluate differences in developing tracheal stenosis with an open versus percutaneous technique.

## Materials and methods

Data source

This was a retrospective review of medical records and was approved as exempt research by Creighton University Institutional Review Board (InfoEd record number: 2003386-01). Patient selection included patients who received an open or percutaneous tracheostomy at Catholic Health Initiatives (CHI) facilities in the Midwest from May 2014 to June 2023. CHI facilities included five facilities in the Omaha metropolitan area and several rural/critical access hospitals. Patients were excluded if they experienced procedural complications requiring a perioperative shift in surgical technique or if they had a pre-existing tracheal stenosis diagnosis.

Aims

The primary aim was to compare the differences in the risk of developing tracheal stenosis between open and percutaneous tracheostomy techniques. For those who developed tracheal stenosis, we aimed to describe the subsite, symptoms, and days required for tracheal stenosis to develop. The secondary aim was to assess differences in risk of other intra- and post-operative complications that included cuff leak, infection, post-operative bleeding, dislodged tube, return to OR for tube size change, intra-operative cardiac issue, mucosal abrasion, and pneumomediastinum secondary to perforation. Although we report individual complication rates, the primary analysis for the secondary aim used composite complications.

Covariates

Descriptives included age, biological sex, race (white, non-white), ethnicity (non-Hispanic/Latino, Hispanic/Latino), prior tobacco use (never, current, former) and pack-year history, comorbid conditions (diabetes, acid reflux or GERD, COPD, asthma, hypertension, obesity), indication for tracheostomy (respiratory failure, trauma/facial fractures, obstruction, neurological injury, vocal cord paralysis, burns), and ventilator status (whether the patient was discharged on a ventilator, history of ventilator dependence, how many days the patient spent on a ventilator). Death and days until death were reported as additional post-operative outcomes.

Statistical analysis

Descriptives were stratified by open vs. percutaneous tracheostomy. Continuous variables were presented as mean and standard deviation or median and interquartile range and compared via t-test or Mann-Whitney U-test, depending on the data distribution. Categorical variables were presented as counts and percentages and compared using a chi-square test or Fischer’s exact test, depending on cell counts.

Between-technique differences in the risk of developing tracheal stenosis were assessed via a Cox proportional hazard model. To account for death precluding patients from developing tracheal stenosis, death was considered a competing risk. Length of follow-up was calculated as the number of days between the tracheostomy procedure and development of tracheal stenosis, or for those who did not experience an event, the number of days between the tracheostomy procedure and the end of the follow-up period (June 2023) or death (if after June 2023).

To estimate the cumulative incidence of tracheal stenosis with death as a competing risk, we used the fine-gray subdistribution hazards model. To estimate the risk of tracheal stenosis after controlling for other factors potentially associated with tracheal stenosis, initial unadjusted Cox proportional hazard models were estimated for covariates that included age, biological sex, race, ethnicity, tobacco use pre- and post-tracheostomy and pack-years smoked, previous ventilator dependence and days spent on a ventilator, whether the patient was discharged on a ventilator following the tracheostomy, comorbid diabetes, GERD/acid reflux, COPD, asthma, hypertension, and obesity. Factors that were statistically associated with the risk of tracheal stenosis were included as covariates in the multivariable model. For all Cox models, the proportional hazards assumption was assessed using scaled Schoenfeld residuals and graphically using log-negative-log survival plots. A Heaviside function was utilized in cases of nonproportional hazards.

Given the absence of time-to-complication data, log-binomial regression models were estimated to assess between-technique differences in the risk of experiencing other intra- and post-operative complications; the log-binomial model did not account for death as a competing risk.

## Results

Descriptives

A total of 828 patients met the inclusion criteria; 542 patients underwent an open tracheostomy and 286 underwent a percutaneous tracheostomy (65.5% vs. 34.5%, respectively). Respiratory failure was the most common indication for tracheostomy (N = 684; 82.6%), followed by trauma/facial fractures (N = 62; 7.5%), neurologic injury (N =48; 5.8%), and obstruction (N = 20; 2.4%). Table 1 presents descriptive characteristics stratified by technique. Briefly, patients who received an open tracheostomy were older (58.1 years vs. 55.6 years; p = 0.035) and more commonly obese (29.3% vs. 17.8%, p < 0.001). However, comorbid GERD/acid reflux was more frequent in patients who received a percutaneous tracheostomy (22.3% vs. 29.0%, p = 0.034).

**Table 1:** Descriptive characteristics stratified by open vs. percutaneous tracheostomy technique Categorical variables were presented as percentages and compared via the chi-square test. Depending on the data distribution, continuous variables were presented as mean ± standard deviation or median (interquartile range) and compared via one-way ANOVA. GERD: gastroesophageal reflux disease; COPD: chronic obstructive pulmonary disease; ANOVA: analysis of variance

		Open	Percutaneous	P-value
Demographic characteristics				
Age (years)		58.10 ± 16.48	55.59 ± 15.76	<0.001
Sex	Male	61.62	61.89	0.941
	Female	38.38	38.11	
Race	White	83.27	81.34	0.499
	Non-White	16.73	18.66	
Ethnicity	Non-Hispanic or Latino	94.51	93.5	0.563
	Hispanic or Latino	5.49	6.5	
Clinical characteristics				
Ventilator status	History of ventilator dependence	82.66	90.56	0.002
	Days on ventilator	9 (4-18)	5 (10-19)	0.084
	Discharged on ventilator	60.7	53.85	0.057
History of tobacco use	Never	42.13	33.82	0.108
	Current	24.72	39.73	
	Former	33.15	30.59	
	Pack-years (current)	15 (7-40)	20 (13-40)	0.34
	Pack-years (former)	21 (10-44)	15 (8-37)	0.147
	Tobacco use following tracheostomy	15.31	22.3	0.014
Comorbid conditions	Diabetes	36.35	31.47	0.161
	GERD or acid reflux	22.32	29.02	0.034
	COPD	18.63	17.83	0.777
	Asthma	11.07	8.04	0.168
	Hypertension	59.78	62.59	0.431
	Obesity	29.34	17.83	<0.001

Primary aim

**Figure 1:**
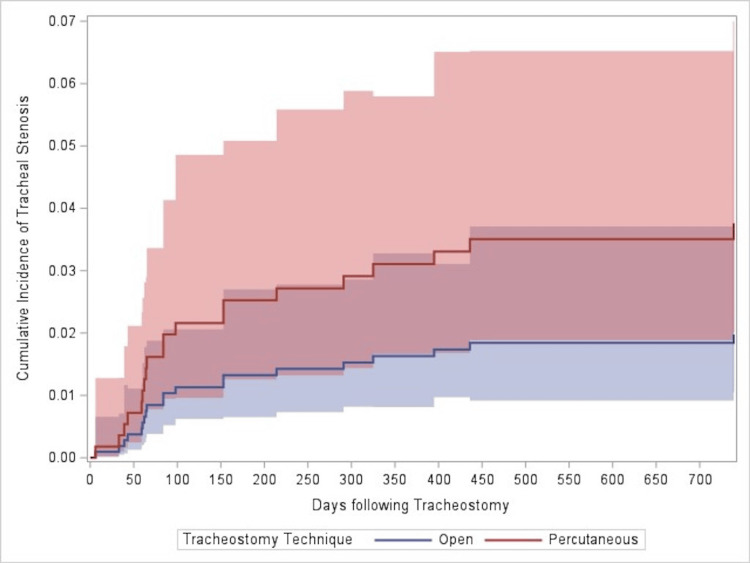
Cumulative incidence of tracheal stenosis stratified by open and percutaneous techniques

Overall, 2.5% (N = 21) of patients developed tracheal stenosis. The median number of days to develop tracheal stenosis was 84 (interquartile range: 60 to 243, range: 6 to 739). The frequency of tracheal stenosis symptoms and subsites is presented in Table 2. Of patients who developed tracheal stenosis, 23.81% (N = 5) had no recorded symptoms, and 33.3% (N = 7) had no recorded subsite. In patients with recorded tracheal stenosis symptoms, dyspnea or shortness of breath was the most common (N = 8; Table 2). In patients with a recorded subsite of tracheal stenosis, subglottic stenosis was the most common (N = 12; Table 2). Tracheal stenosis was more frequent in patients who received a percutaneous tracheostomy (percutaneous: 3.5% vs. open: 2.0%); however, the risk of developing tracheal stenosis was statistically similar between open and percutaneous tracheostomy techniques (HR: 2.05, 95% CI: 0.86-4.94, p = 0.108; Figure 1). No multivariable model was estimated, as all factors that were assessed were not statistically associated with the risk of developing tracheal stenosis (Table 3). Relatedly, the days required for tracheal stenosis to develop were statistically similar between open and percutaneous techniques (p = 0.970). For patients who received an open tracheostomy, the median days to develop tracheal stenosis were 84 (interquartile range: 64 to 214, range: 6 to 395). For patients who received a percutaneous tracheostomy, the median days to develop tracheal stenosis were 108 (interquartile range: 59 to 325, range: 33 to 739).

**Table 2:** Tracheal stenosis symptoms and subsites

	N	%
Symptoms		
Dyspnea/shortness of breath	8	38.1
Respiratory failure	3	14.29
Dysphagia	2	9.52
Dysphonia	1	4.76
Asymptomatic	2	9.52
Not recorded	5	23.81
Subsite		
Subglottic	12	57.14
Suprastomal	1	4.76
Tube cuff/infrastomal	1	4.76
Not recorded	7	33.33

**Table 3:** Cox proportional hazard estimates The reference category is last (i.e., follows "vs."). CI: confidence interval; GERD: gastroesophageal reflux disease; COPD: chronic obstructive pulmonary disease

		Hazard ratio (95% CI)	P-value
Demographic factors			
Age		0.99 (0.96-1.01)	0.356
Sex	Male vs. female	1.57 (0.66-3.78)	0.311
Race	Non-White vs. White	2.48 (0.99-6.21)	0.053
Ethnicity	Hispanic vs. non-Hispanic	0.82 (0.11-6.11)	0.844
Clinical factors			
Ventilator status	Previous dependence (yes vs. no)	0.59 (0.22-1.63)	0.313
	Discharged on a ventilator (yes vs. no)	0.79 (0.33-1.91)	0.602
	Days spent on a ventilator	1.00 (0.999-1.01)	0.1
History of tobacco use	Current vs. never	0.39 (0.08-1.82)	0.23
	Former vs. never	1.83 (0.72-4.63)	0.203
	Pack-years (current)	0.98 (0.85-1.11)	0.708
	Pack-years (former)	1.01 (0.93-1.08)	0.875
	Tobacco use following tracheostomy (yes vs. no)	0.72 (0.21-2.44)	0.593
Comorbid conditions (present vs. absent)	Diabetes	1.17 (0.47-2.92)	0.745
	GERD or acid reflux	0.76 (0.26-2.29)	0.63
	COPD	0.94 (0.27-3.20)	0.937
	Asthma	2.28 (0.76-6.81)	0.141
	Hypertension	1.29 (0.52-3.24)	0.585
	Obesity	2.08 (0.85-5.09)	0.109

Secondary aim

Aside from tracheal stenosis, only 2.7% (N = 22) of patients experienced other intra- or post-operative complications. Bleeding was the most common complication (N = 6; 27.27%), followed by cuff leak (N = 5; 22.73%), infection (N = 4; 18.18%), and dislodged tube (N = 3; 13.64%). Other complications were returned to OR for tube size change (N = 1; 4.55%), mucosal abrasion (N = 1; 4.55%), intra-operative cardiac issue (N = 1; 4.55%), and pneumomediastinum secondary to perforation (N = 1; 4.55%). Notably, these other intra- or post-operative complications were more frequent in patients who received an open tracheostomy (percutaneous: 1.8% vs. open: 3.1%); but similar to tracheal stenosis, the risk of experiencing an intra- or post-operative complication was statistically similar between open and percutaneous approaches (RR = 0.56, 95% CI: 0.21 to 1.50, p = 0.246).

## Discussion

This study found that 2.0% of patients who underwent open tracheostomy developed tracheal stenosis and 3.5% after percutaneous tracheostomy. These rates are consistent with the findings of similar studies, which range from 1.1% to 11.4%, underscoring that tracheal stenosis is an uncommon complication, regardless of the chosen technique [13,20-24]. However, there was no significant difference in the development of tracheal stenosis between different tracheostomy approaches. This aligns with the findings of de Kleijn et al. [24] and Kettunen et al. [23], who also suggested no significant difference, but differs from Maccallum et al. [20] and Hill et al. [21], who suggest a higher incidence of tracheal stenosis with open procedures, and Koitschev et al. [22] and Li et al. [13], who suggest a higher incidence of tracheal stenosis with percutaneous procedures. These studies mostly had smaller sample sizes and varying study focuses, although Kettunen et al. [23] was the most similar study to ours with 616 patients. However, their sample was limited to only trauma patients. Our study recorded tracheostomy for any indication; trauma was the indication in only 7.5% of patients, allowing a more representative population of patients requiring tracheostomy.

Tracheal stenosis can further be categorized by the subsite of the stenosis. Raghuraman et al. [25] found that patients who underwent percutaneous tracheostomy as opposed to open tracheostomy were more likely to develop subglottic stenosis, whereas Koitschev et al. [22] found suprastomal stenosis to occur more in percutaneous tracheostomy. This is significant as there is some evidence of improved outcomes with subglottic or stoma subsite stenosis versus cuff or tip site stenosis [26]. The majority of patients in both groups of our study demonstrated subglottic stenosis, with only one reported instance of suprastomal stenosis in an open technique and one distal tip stenosis in the percutaneous technique. Additionally, Raghuraman et al. [25] found stenosis in percutaneous tracheostomy occurred earlier than in open tracheostomy. This is consistent with our study, which demonstrates a longer median time to develop tracheal stenosis with open tracheostomy at 84 days versus percutaneous tracheostomy at 108 days, although no statistically significant difference was found.

It is well known that race and ethnicity act as social determinants of health [27]. Limited studies have looked specifically at demographic factors such as race and ethnicity on the development of tracheal stenosis, although Johnson and Saadeh [10] and Dang et al. [28] both found no significant difference. We similarly report no significant difference between race and ethnicity in the development of tracheal stenosis.

Prior studies have demonstrated an increased risk of tracheal stenosis with prolonged mechanical ventilation [12-14]. This is primarily demonstrated in the setting of ventilation in orotracheal intubation, which often presents as the indication for performing a tracheostomy. However, increasing time on mechanical ventilation with a tracheostomy, leading to an increased risk of tracheal stenosis, has not been elucidated. Our study did demonstrate an overall increased risk of developing tracheal stenosis if the patient had ventilation dependence prior to tracheostomy, but no significant difference was seen with increased time on mechanical ventilation after tracheostomy and the development of tracheal stenosis.

Various comorbidities and factors that increase the risk of developing tracheal stenosis have been described in the literature. An increased risk of developing tracheal stenosis was described by Koizumi et al. [18] in female patients, those with a low BMI, or those who use long-term mechanical ventilation, whereas Li et al. [13] found stenosis occurred more in patients with obesity. Burruss et al. [18] found stenosis occurred more with increasing age and BMI. Various studies have additionally found associations in comorbid conditions such as GERD, hypertension, diabetes mellitus, and COPD [8,15,16]. Further, tracheal stenosis development has been associated with smoking [8,16]. However, many of these associations have not been demonstrated consistently across studies. Our results demonstrated that the risk of tracheal stenosis was statistically similar across age, sex, prior or current tobacco use, and comorbid conditions including obesity, diabetes, acid reflux or GERD, COPD, asthma, and hypertension.

Given the relatively low incidence of tracheal stenosis and the lack of significant differences between techniques, this study suggests tracheal stenosis should not be a determining factor in deciding between open versus percutaneous techniques. Other intra- and post-operative complication rates also demonstrate no significant difference and suggest no influence on clinical decision-making. Despite their increasing popularity, there are still contraindications to percutaneous tracheostomies. These include patients with morbid obesity, difficulty identifying the trachea, and an inability to safely extend the neck due to conditions like cervical instability or distortion of normal anatomy [1,29]. Therefore, these are the types of patients who should be considered for an open approach. Outside of these contraindications, surgical techniques should be selected based on surgeon and patient preference, along with the clinical situation.

This study does have limitations to address. Although the overall sample size is larger than prior similar studies, given the low rates of tracheal stenosis, there are still only 21 patients who developed tracheal stenosis to analyze. Additionally, although a strength of our study is having a longer follow-up period than existing literature, there is still a possibility of missing tracheal stenosis development based on limitations in follow-up. Patient charts were reviewed for any development of tracheal stenosis from the time of their tracheostomy up until the point chart review was done. Therefore, patients who received a tracheostomy later had a shorter follow-up period. However, all patients had at least six months of follow-up. Most commonly, tracheal stenosis takes weeks to develop, with reported averages ranging from 10 to 41 days [11,30]. Our study average was 84 days. Although it would take an unusually long time to develop tracheal stenosis, there is a chance that patients could develop tracheal stenosis after the chart review is completed. Additionally, our chart review is limited by follow-up within facilities included in our electronic health record. There is the potential that patients could have developed tracheal stenosis but presented to an outside institution. Additionally, tracheal stenosis can be subclinical, and many patients can be asymptomatic. Therefore, it is possible for patients who have developed tracheal stenosis but have not been symptomatic to seek out care and receive a diagnosis. Finally, the retrospective nature of our review led to inconsistent reporting in the subsite of tracheal stenosis, limiting our ability to analyze between-technique differences. Given these limitations, additional studies are needed to further elucidate this question, particularly prospective studies.

## Conclusions

This study demonstrates no significant difference in the development of tracheal stenosis when performing an open versus a percutaneous tracheostomy. Tracheal stenosis is a long-term complication of tracheostomy and should not influence the decision about the surgical technique used.
